# Transcriptome and gene expression analysis of DHA producer *Aurantiochytrium* under low temperature conditions

**DOI:** 10.1038/srep14446

**Published:** 2015-09-25

**Authors:** Zengxin Ma, Yanzhen Tan, Guzhen Cui, Yingang Feng, Qiu Cui, Xiaojin Song

**Affiliations:** 1 Shandong Provincial Key Laboratory of Energy Genetics, Qingdao Institute of Bioenergy and Bioprocess Technology, Chinese Academy of Sciences, Qingdao, Shandong, China; 2 Key Laboratory of Biofuels, Qingdao Institute of Bioenergy and Bioprocess Technology, Chinese Academy of Sciences, Qingdao, Shandong, China; 3 Qingdao Engineering Laboratory of Single Cell Oil, Qingdao, Shandong, China; 4 University of Chinese Academy of Sciences, Beijing, China

## Abstract

*Aurantiochytrium* is a promising docosahexaenoic acid (DHA) production candidate due to its fast growth rate and high proportions of lipid and DHA content. In this study, high-throughput RNA sequencing technology was employed to explore the acclimatization of this DHA producer under cold stress at the transcriptional level. The overall *de novo* assembly of the cDNA sequence data generated 29,783 unigenes, with an average length of 1,200 bp. In total, 13,245 unigenes were annotated in at least one database. A comparative genomic analysis between normal conditions and cold stress revealed that 2,013 genes were differentially expressed during the growth stage, while 2,071 genes were differentially expressed during the lipid accumulation stage. Further functional categorization and analyses showed some differentially expressed genes were involved in processes crucial to cold acclimation, such as signal transduction, cellular component biogenesis, and carbohydrate and lipid metabolism. A brief survey of the transcripts obtained in response to cold stress underlines the survival strategy of *Aurantiochytrium*; of these transcripts, many directly or indirectly influence the lipid composition. This is the first study to perform a transcriptomic analysis of the *Aurantiochytrium* under low temperature conditions. Our results will help to enhance DHA production by *Aurantiochytrium* in the future.

Docosahexaenoic acid (DHA; C22:6, ω-3) is one of the most important polyunsaturated fatty acids (PUFAs) in humans, as it widely exists in human neural and retinal tissues^1^. DHA has drawn increasing amounts of attention due to its beneficial effects in the cognitive development of infants^2^,^3^ and its reduction of the risk of hypertension, cardiovascular diseases, inflammation and certain cancers^4^,^5^. Currently, the major commercial source of DHA is fish oil, which contains 7–14% DHA^6^. However, some drawbacks, including marine contaminants in fish, undesirable fishy flavor, and the oxidative instability of fish oil, make alternative sources of DHA increasingly attractive^7^. Marine microorganism species such as *Aurantiochytrium* sp.^8^ and *Crypthecodinium cohnii*^9^ have become the major alternative sources for commercial DHA production.

*Aurantiochytrium* sp. are heterotrophic marine thraustochytrids that are ubiquitous to marine and estuarine waters^10^. They have a greater ability to produce biomass and DHA yields compared to *C. cohnii*^11^. Moreover, *Aurantiochytrium* can produce large amounts of lipids, which comprise up to 60% of the dry cell weight; DHA comprises nearly 40% of the total fatty acid content^12^. During recent decades, many culture optimization strategies, such as aeration, pH adjustment, temperature variation, optimization of nutrient media components, and so on have been performed^13^,^14^. Various environmental conditions have an effect on the biomass, lipid productivity and fatty acid composition, and temperature is thought to be one of the most important factors. Many researchers have found that low temperatures can limit cell growth but can increase the proportion of unsaturated fatty acids produced, including DHA^15^,^16^. It is widely accepted that these changes are aimed at maintaining proper membrane fluidity and function, but the specific mechanism still remains unclear.

With the emergence of next-generation sequencing (NGS) technology, high throughput sequence determination has dramatically improved the speed of gene discovery and the sensitivity of gene expression profiling^17^, and can be used for functional, comparative and evolutionary genomics studies^18^. To date, the transcriptomes of a large number of oleaginous microorganisms, including *Neochloris oleoabundans*^19^, *Yarrowia lipolytica*^20^, and *Nitzschia*^21^, have been analyzed using NGS.

In this study, we used Illumina’s sequencing technology to examine global changes in the *Aurantiochytrium* sp. SD116 transcriptome in response to low temperature stress for the first time, contributing to a better understanding of the various functions of genes that are differentially expressed under cold stress. The results provide an important resource for future genetic engineering studies aimed at improving DHA production, providing new insight into the regulation of lipid metabolism in *Aurantiochytrium* at the transcriptomic level.

## Results

### Growth and glucose consumption curve

**Figure 1:**
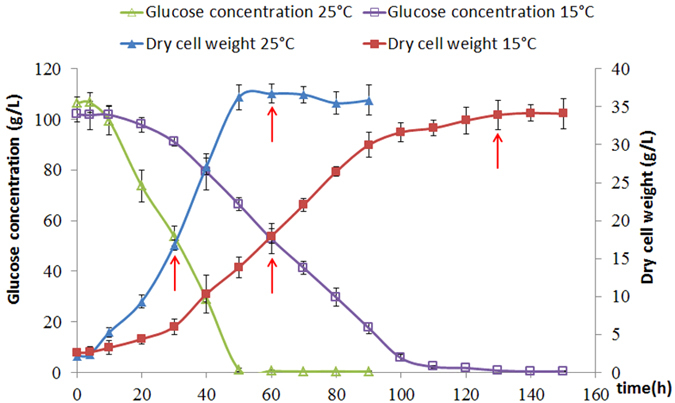
Growth curve and glucose consumption at 25 °C and 15 °C.

Figure 1 shows that *Aurantiochytrium* grew faster at 25 °C than at 15 °C. At 25 °C, cells quickly reached the logarithmic phase at approximately 10 h and the stationary phase at 50 h, with a maximum biomass of approximately 36 g L^−1^. At 15 °C, the cells reached the logarithmic phase at 30 h and the stationary phase after 120 h of cultivation. The final biomass was slightly lower at the lower temperature than at the normal temperature (approximately 34 g L^−1^). The changes in glucose concentration essentially reflected the cell growth. The glucose consumption rate was higher in the normal temperature group (25 °C) compared with the low temperature group (15 °C). After the stationary phase, the glucose in the medium was completely consumed in both groups.

### Impact of cold induction on the fatty acid composition of *Aurantiochytrium* sp. SD116

To detect the changes in the fatty acid composition caused by cold stress, lipid accumulation was quantitatively examined via the transesterification of glycerolipids into fatty acid methyl ester (FAME), and significant differences were observed between the low temperature and control groups (Table 1). In the logarithmic phase, the cells contained nearly 25% lipids on a dry cell weight basis, and lipids accumulated to over 50% in the stationary phase. Both groups had similar lipid content in the growth stage, but the fatty acid composition, especially the amount of PUFAs (DPA and DHA) and palmitic acid (C16:0), was remarkably different after the accumulation of lipids in cells. In the normal temperature groups, the DHA and DPA content decreased slightly, while the saturated fatty acid (C16:0) content increased from 40.5% to 43.5%. During low temperature fermentation, the palmitic acid was reduced from 38% to 33%, whereas DHA and DPA accumulated from 47.6% to 52.5% and from 8.27% to 9.27%, respectively. These results indicate a phenomenon reflecting a cold acclimation mechanism and are in agreement with previous conclusions^22^,^23^. Many studies^24^,^25^ have confirmed that the accumulation of unsaturated fatty acids can improve membrane fluidity and therefore survivability under cold circumstances.

### Identification of expressed transcripts in the *Aurantiochytrium* sp. SD116 transcriptome

**Figure 2:**
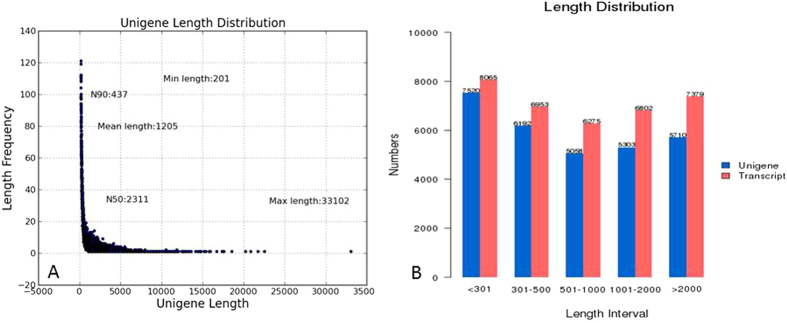
(**A**) Unigene length distribution. (**B**) Unigene and transcript length distribution.

To obtain an overview of the *Aurantiochytrium* sp. SD116 gene expression profiles at different temperatures, we sequenced a cDNA library prepared from total mRNA using Illumina technology. Overall, there were approximately 26 to 39 million raw reads for each sample (Table 2). After removing the adaptor sequences, ambiguous nucleotides, and low-quality sequences, approximately thirty thousand transcripts were obtained from all samples, with an average length of 1,200 bp and length ranging from 201 to 33,102 bp. The longest copy of a set of redundant transcripts was regarded as a unigene, and a total of 29,783 unigenes were identified (Fig. 2).

**Figure 3:**
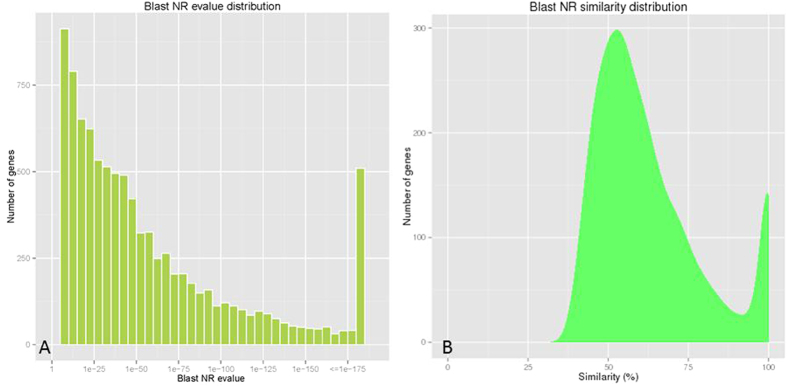
(**A**) E-value distribution of BLAST hits for unigenes with a cutoff E-value of 1.0E-5. (**B**) Similarity distribution of the top BLAST hits for unigenes.

The generated transcripts were queried against seven different public databases with an E-value threshold of 10^−5^ (1.0E-5) for the similarity search (Fig. 3). In total, 13,245 (44.47%) unigenes were annotated in at least one database, while 980 (3.29%) unigenes shared similarity in all seven databases. Unfortunately, there was still 16,538 (55.53%) undefined unigenes (Table 3). The E-values and score distributions of the best hits in the Nr database revealed that 38% (3,504) of the matched sequences were highly homologous, with a score >500 and 41% (3,779) and an E-value < 1E-50.

### Functional classification of unigenes

**Figure 4:**
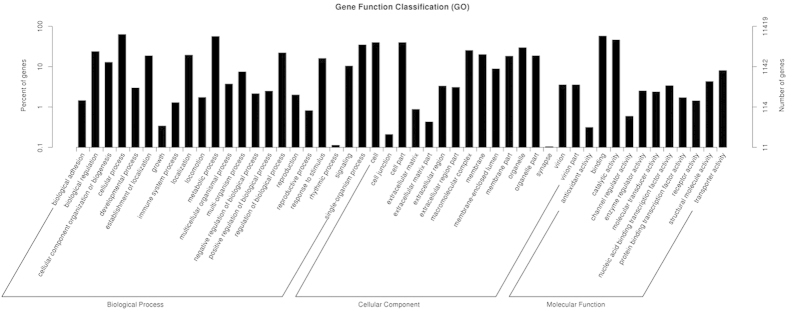
GO classifications of assembled unigenes.

Gene Ontology (GO) is an international standardized gene functional classification system that defines genes according to three ontologies: molecular function, cellular component and biological process. In this test, we annotated 11,419 unigenes (38.34%) to the GO database. These unigenes were assigned to 49 subcategories (Fig. 4). Among these unigenes, a majority were classified as biological process (34,962, 46.99%), followed by cellular part (24,736, 33.24%) and molecular function (14,709, 19.77%). In the biological process category, the major subcategories were “cellular process” (7,264, 20.78%) and “metabolic process” (6,484, 18.54%). In the cellular component subcategory, the largest proportion subcategories consisted of both “cell” (4,559, 18.43%) and “cell part” (4555, 18.41%), followed by “organelle” (3,406, 13.77%). In the molecular function category, a significant proportion of the clusters were classified as “binding” (6,595, 44.84%) and “catalytic activity” (5,270, 35.83%). Moreover, within each of the three categories, a few unigenes were assigned to the “rhythmic process”, “cell junction”, “synapse” or “translation regulator activity” categories.

**Figure 5:**
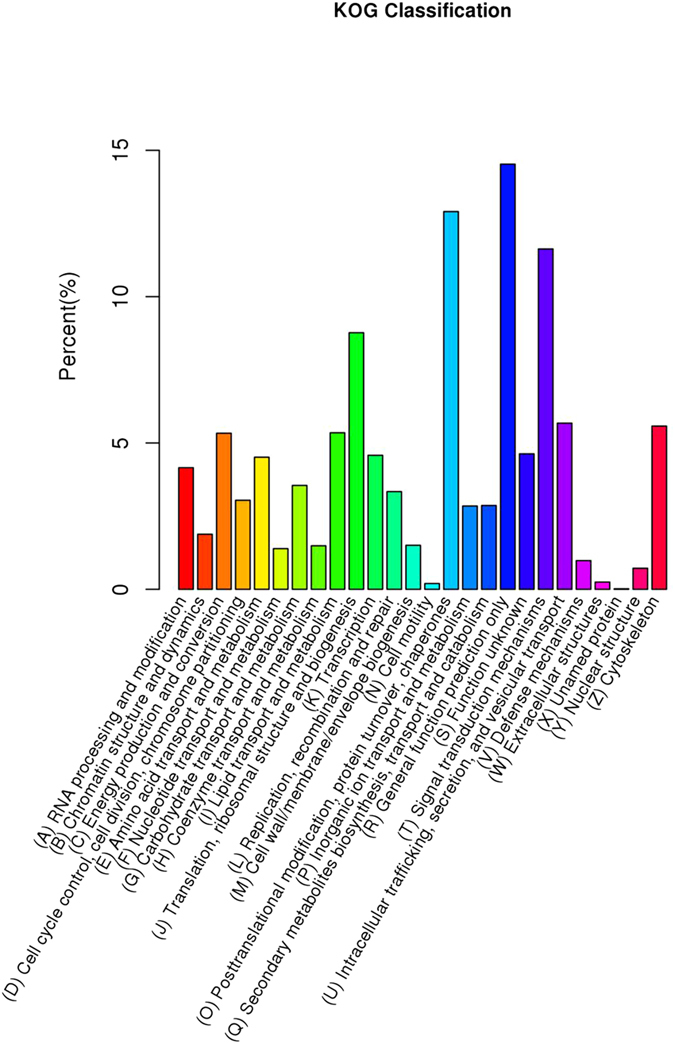
KOG/COG classification of unigenes.

The functions of the unigenes were further classified according to the COG/KOG databases, which are used to classify orthologous gene products. Through KOG analysis, a total of 6,113 (20.52%) non-redundant unigenes were divided into 26 KOG classifications (Fig. 5). In comparison, most of the unigenes were categorized as “General function prediction” (888, 13%), followed by “Post-translational modification, protein turnover, and chaperone” (789, 11.55%), and “Signal transduction mechanisms” (711, 10.41%). Except for “unnamed protein”, the smallest group was “cell motility” (12, 0.18%).

**Figure 6:**
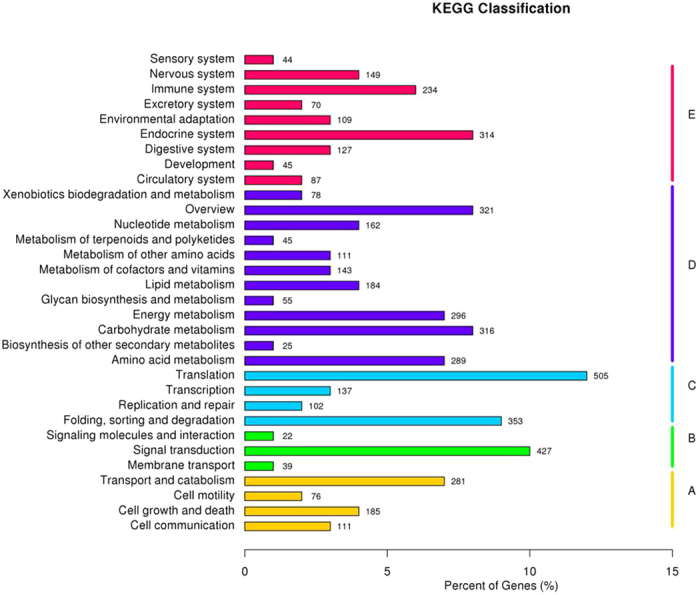
KEGG classification of unigenes. The capital letters against the colored bars indicate five main categories: (**A**) cellular processes; (**B**) environmental information processing; (**C**) genetic information processing; **(D**) metabolism; and (**E**) organism systems.

The orientations of the unigenes in metabolic pathways were analyzed by querying the KEGG database. In total, 4,116 unigenes were assigned to five specific pathways, including cellular processes, environmental information processing, genetic information processing, metabolism and organismal systems (Fig. 6). These five pathways were further assigned to 32 KEGG pathways, in which the main metabolism terms were “translation”, “signal transduction” and “folding, sorting and degradation”. 184 unigenes (4.47%) were classified as “Lipid metabolism”, which we considered most important.

### Low temperature induced changes in the transcriptional profile of *Aurantiochytrium* sp. SD116

To better understand the biological mechanism resulting in transcriptomic changes in *Aurantiochytrium* incubated at a low temperature (15 °C), it was important to identify the differentially expressed (DE) genes between normal and low temperatures. We separately calculated and analyzed the DE genes of *Aurantiochytrium* in the logarithmic and stationary phases at two temperatures (15 °C vs 25 °C, q-value < 0.05 & |log2 Fold change| > 1) and found that there were 2,013 DE genes (1,259 upregulated and 754 downregulated) in the logarithmic phase and 2,071 DE genes (1,235 upregulated and 836 downregulated) in the stationary phase. Notably, 573 genes were significantly differentially expressed in both phases. Of these, 317 genes were upregulated. Most of these genes were hypothesized to participate in the cold response and resistance. These include transcription factors, ATP-dependent RNA helicase, and signal transduction-related genes, such as G protein signaling regulators, Ca^2+^/calmodulin-dependent protein kinase, and more.

The genes identified as differentially expressed were further classified into 26 sub-functional categories according to the KOG classification principles (Table 4). Many metabolic processes were found to be involved in low temperature induction, and some functional categories were particularly changed. In general, there were two adverse variation trends according to the major DE genes in these sub-categories. Most metabolic processes involved in the biogenesis of cellular components, the cell cycle, and signal transduction had more upregulated genes in the logarithmic phase. In most cases, the number of DE genes in these processes obviously decreased during the stationary phase, at which point, the overwhelming majority of these genes were downregulated. This type of gene expression change reflected the adaptive mechanism to cold stress. Many specific cold-shock proteins, which are involved in transcription, translation and other fundamental functions and play a role in maintaining nucleic acid structure and growth under cold conditions, were induced^26^. Only biological processes related to energy production and carbohydrate metabolism showed another type of variation trend: many more of these genes were upregulated during the stationary phase. DE genes involved in lipid transport and metabolism were slightly upregulated in both phases.

### Changes in DE gene profiles induced by low temperature during lipid accumulation stages

In oleaginous microorganisms, lipid content can accumulate to more than 50% of the total biomass, as measured in dry weight. This mainly occurs near the stationary phase of fermentation. During this period, nitrogen exhaustion leads to the cessation of cell growth, and surplus carbohydrates are mainly transformed into lipid deposits. To further understand lipid accumulation changes caused by low temperatures, we also observed the changes in DE gene expression profiles during the lipid accumulation stage (130 h at 15 °C). On the whole, there were 5,352 DE genes (2,363 upregulated and 2,989 downregulated) in the low temperature group during the lipid accumulation stages (stationary phase vs logarithmic phase). DE genes were also classified according to the KOG database (Table 4); some biological processes were found to be significantly affected by low temperatures during the lipid accumulation stage. These processes included signal transduction mechanisms; posttranslational modification, protein turnover, chaperones; lipid transport and metabolism; energy production and conversion; amino acid transport and metabolism; and cytoskeleton and carbohydrate transport and metabolism. In each biological process, over 60 genes were differentially expressed.

### Validation of gene expression profiles by qRT-PCR

To validate the RNA-seq data, qRT-PCR was performed for 10 genes, most of which were associated with lipid and fatty acid biosynthesis. A single product was amplified according to each melting curve analysis. Except diacylglycerol acyltransferase, acetyl-CoA carboxylase, and fatty acid synthase, which had opposite results in a few comparison groups, most genes showed similar changes in expression levels, indicating that the RNA-seq data were reliable (Table 5, Fig S1).

### Differentially Expressed Genes Related to Fatty Acid Synthesis

As *Aurantiochytrium* is a predominantly oleaginous microorganism, we paid particular attention to differentially expressed genes related to fatty acid synthesis (Table 5). Fatty acid synthase and polyunsaturated fatty acid synthase are separately responsible for producing saturated and polyunsaturated fatty acid. In the logarithmic phase, low temperatures led to the separately downregulated expression of the fatty acid synthase and three polyunsaturated fatty acid synthase subunits by approximately 3.7-fold and 2-fold, respectively. Although the Q-PCR and RNA-seq results indicated that during the stationary phase, fatty acid synthase had some opposite changes in its differential expression, most results indicated that this change was less than 1.7-fold. In contrast, all of the polyunsaturated fatty acid synthase subunits were upregulated by approximately 3-fold in the stationary phase, which is the major stage of lipid accumulation. Under cold stress, Acetyl-CoA carboxylase, which provides the carbon skeleton in the fatty acid biosynthesis pathway, was separately downregulated by 2.5-fold during the logarithmic phase and was upregulated by 2-fold in the stationary phase. Further experimental evidence is required to explain the inconsistency between the Q-PCR and RNA-seq results. Fatty acid synthesis requires a large amount of reducing power (NADPH); glucose-6-phosphate 1-dehydrogenase and malic enzyme are both major producers of NADPH in different metabolite pathways. At the low temperature, malic enzyme was separately downregulated by nearly 2-fold and 1.3-fold in the logarithmic and stationary phases, respectively. In contrast, glucose-6-phosphate 1-dehydrogenase was only downregulated by 1.2-fold during the logarithmic phase. Though different enzymes act to synthesize saturated and polyunsaturated fatty acids, further evidence is needed to determine whether different enzymes provide the reducing power for these separate synthesis processes.

During the stationary phase, there was little nitrogen in the media, and the cells accumulated large amounts of lipids. We found that relative to the logarithmic phase, fatty acid synthase during the stationary phase was downregulated by 3.4-fold and 1.2-fold at 25 °C and 15 °C, respectively. All polyunsaturated fatty acid synthase subunits were sharply downregulated by more than 100-fold at both temperatures. Particularly in the normal temperature group, the expression of polyunsaturated fatty acid synthase subunits declined by more than 1000-fold. In addition to these genes, the expression of acetyl-CoA carboxylase declined by 3.6-fold at 15 °C and 18-fold at 25 °C. The genes involved in the production of reducing power (NADPH), including malic enzyme, glucose-6-phosphate 1-dehydrogenase and isocitrate dehydrogenase, all showed varying degrees of reduced expression levels at both temperatures. Under nitrogen limitation, the cells downregulated the expression of genes related to fatty acid synthesis but upregulated a key gene in the TAG synthesis pathway, diacylglycerol acyltransferase (DGAT), by nearly 2-fold at both temperatures. We speculated that the fatty acid synthesis-related enzymes expressed during the logarithmic phase were sufficient to facilitate lipid accumulation in the stationary phase, or perhaps the cells used other means of regulating the activities of these enzymes during this stage. Nonetheless, the level of DGAT expression is essential for TAG synthesis during the stationary phase. Thus, this may be an important genetic engineering target and requires further confirmation.

## Discussion

*Aurantiochytrium* can accumulate large amounts of lipid under appropriate fermentation conditions, and DHA (C22:6) comprises over 40% of the total fatty acid amount, making *Aurantiochytrium* a promising organism for sustainable DHA production. Many studies^6^,^16^ have confirmed that low temperatures increased the DHA content to some degree, but the specific mechanism has yet to be elucidated. In this study, we used next-generation sequencing techniques to analyze the global differential gene expression profiles between normal and low temperatures, finding that the DE genes were related to cold acclimation (All the genes mentioned in this paper were listed in Table S1). This result provides a broad overview and can be used to guide future studies aimed at improving lipid and DHA accumulation in *Aurantiochytrium*.

In *Aurantiochytrium*, there are two fatty acid synthesis pathways. The type I fatty acid synthase (FAS) pathway produces saturated fatty acids, which mainly consist of palmitic acid (C16:0), while the polyketide synthase-like polyunsaturated fatty acid synthase (PKS) pathway is involved in DHA (C22:6) and DPA (C22:5) synthesis^27^,^28^. In the current study, the expression of PUFA synthases (Pufa A/B/C) remarkably increased under conditions of cold stress, whereas the type I fatty acid synthase showed no significant differential expression. This may explain the changes in the fatty acid compositions at low temperatures: unsaturated fatty acid chains introduce more disturbance into the cell membrane than saturated fatty acid chains^29^, thereby increasing the fluidity of the membrane. *Photobacterium profundum*^30^ and *Shewanella piezotlerans*^31^ also use PKS pathway to synthesize PUFA, but at reduced temperatures, the expression of PUFA genes did not increase, despite the increased percentage of PUFAs in the total fatty acid content. The inconsistency in these results indicated that different mechanisms of regulating PUFA synthesis exist in these organisms, which may be related to the discrepancy in their evolutionary statuses. Acetyl-CoA carboxylase (ACC), which produces malonyl-CoA for use in both the FAS and PKS pathways, is another key enzyme in the fatty acid biosynthesis pathway^32^. Cold induction resulted in low ACC expression at the beginning of the experiment but overexpression at the end, providing more malonyl-CoA for fatty acid synthesis. Notably, more malonyl-CoA is required for the synthesis of PUFAs than the synthesis of saturated fatty acids according to their carbon chain lengths. Luis *et al*.^33^ also found that in *Chlamydomonas reinhardtii*, ACC was upregulated both at the gene and protein levels under cold stress. The long–chain acyl-CoA synthetase (ACSL) activates long-chain and very-long-chain fatty acids to form acyl-CoAs and channels fatty acids into different pathways of the complex lipid metabolism, including the TAG and phospholipid biosynthesis pathways and the fatty acid degradation pathway^34^. Because we found this gene to be overexpressed by more than 2.5-fold times under cold stress, we speculated that much more acyl-CoA was used in lipid biosynthesis. The expression of key enzymes that aid the transport of fatty acids into the mitochondria for beta oxidation, carnitine o-acyltransferase *CPT1* and carnitine o-acyltransferase *CPT2* decreased by 1.5-fold and 3.5-fold over the same time period. Cheng *et al*.^21^ found that ACSL was significantly upregulated in oleaginous diatoms under conditions of environment stress. In our study, though diacylglycerol acyltransferase (DGAT) was obviously downregulated by 2.3-fold, the expression of genes related to phospholipid synthesis increased by varying degrees. The expression of lysophosphatidic acid acyltransferase (LPAAT) for phosphatidic acid biosynthesis increased by 4-fold, and the expression of phosphatidylinositol transfer protein (PITP), which transports the phospholipids from their site of synthesis to the cell membranes^35^, increased by 2-fold. The expression of the phospholipid degradation enzyme phospholipase, however, decreased by 1.7-fold. These results indicated that low temperatures contributed to the accumulation of phospholipids and the restriction of TAG synthesis. The fluidity of the membrane is altered much more effectively by changes in the fatty acid structures of phospholipids during low temperature growth^36^. Moreover, during the stationary phase under low temperature conditions, many genes involved in sterol biosynthesis, including sterol 24-C-methyltransferase, cycloartenol synthase, cholesterol transport protein and squalene synthetase, obviously increased by at least 2-fold. Sterols are essential lipids of most eukaryotic cells that ensure important structural and signaling functions. Ergosterol is the main sterol found in most fungi and is the end-product of a long, multistep biosynthetic pathway that converts squalene into its downstream product^37^. Jordi *et al*.^38^ found that *Saccharomyces cerevisiae* showed a general increase in the amounts of sterol esters and squalene when cultured at low temperatures. Marian *et al*.^39^ also demonstrated that *Saccharomyces cerevisiae* with a higher ergosterol content showed higher viability at low temperature. In *Arabidopsis*, sterol synthesis was upregulated during the late response of cold acclimation^40^, which was a similar finding to our data. In summary, *Aurantiochytrium* accommodated the cold environment by adjusting its composition of fatty acids and its lipids metabolism during different phases.

Apart from changes in the lipid composition, the perception and transduction of low-temperature signals is another immediate acclimation to cold stress. Histidine kinases are multifunctional, transmembrane proteins that play vital roles in signal transduction across the cellular membrane^41^. In *Synechocystis*, histidine kinase appears to be involved in the perception and transduction of low-temperature signals that regulate the low temperature-dependent induction of the lipid desaturase and RNA helicase genes^42^. In our analysis of the transcriptome, this gene was upregulated by more than 4-fold during the stationary phase of cold induction, although its expression slightly decreased during the logarithmic phase. This result indicated that histidine kinase may not an immediate signal transduction enzyme in the cold stress response of *Aurantiochytrium*. In *Saccharomyces cerevisiae*, the cAMP-PKA pathway plays a major role in controlling the genetic responses to a wide variety of stresses. For instance, Ser/Thr/Tyr protein kinases are involved in the cold-shock signaling pathway occurring through PKA^26^. Our results confirmed that both tyrosine kinase and cAMP-dependent protein kinase were overexpressed, especially during the early stages of cold response. In yeast and animals, mitogen-activated protein kinase (MAPK) pathways are activated by receptors such as protein tyrosine kinases, G-protein-coupled receptors, and two-component histidine kinases^43^. These pathways are responsible for the production of cold-compatible osmolytes and antioxidants, which aid the organism in resisting the adverse environmental conditions. Except for the previously mentioned tyrosine/histidine kinases, we found that G protein signaling regulators were also overexpressed in both cold stress processes. Except for the differential expression of protein kinases, cold stress also induced transient Ca^2+^ influx into the cell cytoplasm. The activation of certain Ca^2+^ channels by cold may result from physical alterations in cellular structures^43^. We observed that Ca^2+^ sensors and Ca^2+^/calmodulin-dependent protein kinases mostly increased in expression during the logarithmic phase, but decreased or were not obviously changed during the stationary phase, indicating that these genes may be involved in an upstream signal transduction pathway during cold stress. As second messengers, diacylglycerol (DAG) and IP_3_ (inositol 1,4,5-trisphosphate) also have inextricable relationships with Ca^2+^ release. IP_3_ is produced from the hydrolysis of phosphatidylinositol 4,5-bisphosphate (PIP_2_), which is activated by the signaling pathways initiated by the major lipid product of phosphoinositide 3-kinase (PI3K)^44^. The catalysis of DAG into phosphatidic acid (PA) by diacylglycerol kinase (DGK) requires ATP as a source of the phosphate. We found that the generation of PA through DGK controls the phosphoinositide (PI) cycle via direct recycling into PIP_2_^45^. Therefore, DGK occupies an important position in the adaptive cold response. As key components of this signal pathway, PI3K and DGK were all overexpressed to different degrees during the early stages of cold stress. These differentially expressed signaling pathway genes may further activate other metabolic processes in the cell to facilitate acclimatization to ambient conditions. Further research is needed to determine which of these genes are related to the activation of the lipid or PUFA synthesis pathways.

It is accepted that decreases in environmental temperature are closely related to increased levels of intracellular oxidative stress, which leads to metabolic rearrangements that bypass enzymes that provide necessary reducing agents^46^. To better understand the diversion of metabolic flux under cold stress, we compared the transcript abundances of the genes encoding enzymes involved in the central carbon metabolism pathways, including glycolysis, the TCA cycle and the pentose phosphate pathway. Hexokinase (HK) catalyzes the first step in the glycolytic pathway, phosphorylating glucose to form glucose-6-phosphate. Our data showed that HK was downregulated by 1.7-fold and 1.2-fold during the logarithmic and stationary phases, respectively. Nedelina *et al*.^47^ found that short-term exposure to cold stress led to a general decrease in the activity of HK in *Penicillium* sp. and *Aspergillus glaucus*; these authors attributed these results to the inhibition of HK by the accumulation of trehalose in the cytosol at low temperatures as a stress protectant. However, we did not find evidence of changes in trehalose synthesis, though this finding needs further experimental verification. Phosphofructokinase (PFK) catalyzes the ATP-dependent phosphorylation of fructose-6-phosphate into fructose 1,6-bisphosphate in one of the key regulatory and rate-limiting steps of glycolysis. In this study, during the logarithmic phase, the expression of PFK transcripts decreased by 1.5-fold. However, its reverse reaction, which is catalyzed by fructose 1,6-bisphosphatase in the gluconeogenesis pathway, increased by 1.3-fold during the logarithmic phase. Another key enzyme in glycolysis, glyceraldehyde 3-phosphate dehydrogenase (GAPDH), was overexpressed by 2.6-fold and 1.8-fold separately during the logarithmic phase and stationary phase, which was in agreement with Nedelina’s results^47^. Upregulation of GAPDH transcripts could be explained by its importance in stress conditions leading glycolytic flux deviation to accumulation of reserve carbohydrates^48^. These results reflected that the glycolysis pathway was inhibited to some extent under cold stress. The pentose phosphate pathway (PPP) is a biochemical pathway parallel to glycolysis that generates NADPH and pentose. Many studies have indicated that the key enzymes in the PPP pathway, glucose-6-phosphate dehydrogenase (G6PD) and 6-phosphogluconate dehydrogenase (PGD), are overexpressed under different adverse conditions such as high-salinity stress^21^, cold stress^33^,^47^ and oxidative stress^49^ to maintain the balance of NADPH. Our results showed that under cold stress, the expression of G6PD only decreased by 1.2-fold during the logarithmic phase, whereas PGD expression increased by 1.3-fold during the stationary phase. However, two downstream enzymes in the PPP pathway, ribokinase and ribose 5-phosphate isomerase, were both obviously overexpressed by more than 1.5-fold during the stationary phase. Whether cold stress leads to the overexpression of PPP pathway components in *Aurantiochytrium* needs further verification. When we evaluated the TCA cycle, we found that two parts of the cycle showed differential expression patterns. Most enzymes in the latter part of the cycle, including succinyl-CoA synthetase and fumarate hydratase, were obviously downregulated by more than 1.6-fold. By contrast, most of the enzymes in the first half of the cycle, including citrate synthase and isocitrate dehydrogenase (ICDH), showed no significant changes. Martin *et al*.^50^ obtained similar results in *Arabidopsis* under conditions of oxidative stress. In that study, the metabolites in the non-decarboxylating part of the TCA cycle, including succinate, fumarate and malate, were both decreased. In consideration of glycolysis inhibition during this period, more glucose was probably re-routed into the PPP pathway, resulting in greatly increased reducing power to mitigate the cold stress as well as ribose for ribosome synthesis.

## Conclusions

In this study, we used next-generation sequencing techniques to characterize the transcriptome and acquire the gene expression profiling data of *Aurantiochytrium* sp., a promising candidate for DHA production. By comparing the differential gene expression profiles under normal and low temperatures, we found that a substantial number of genes have potential roles in the adaptation to cold environments. Of these genes, many have direct or indirect influences on cellular lipid composition. This is the first study to perform a transcriptomic analysis of the DHA producer *Aurantiochytrium* under low temperature conditions. Our results enrich the current knowledge regarding the metabolic functions of *Aurantiochytrium* under conditions of cold stress, and our findings will help to enhance the production of DHA by *Aurantiochytrium* in the future.

## Methods

### Strain and culture conditions

*Aurantiochytrium* sp. SD116 was cultured under the same conditions as in our previous study^51^. The seed culture medium contained 60 g L^−1^ glucose, 20 g L^−1^ yeast extract and 15 g L^−1^ artificial seawater. Batch cultures were performed in 5 L Biostat B plus bioreactors in medium containing 100 g L^−1^ glucose, 20 g L^−1^ yeast extract, 10 g L^−1^ tryptone, 5 g L^−1^ KH_2_PO_4_ and 15 g L^−1^ artificial seawater. The fermentation temperatures were separately maintained at 25 °C (control group) and 15 °C (cold induction group).

### Biomass and glucose assay

The *Aurantiochytrium* sp. biomass was expressed in terms of dry cell weight (DCW). 10-mL samples of cell suspensions were centrifuged at 7,000 × *g* and 4 °C for 10 min after washing twice with 0.2 M phosphate buffer. The cell pellets were then freeze-dried to a constant weight at −50 °C for approximately 40 h. The residual glucose in the fermentation medium was analyzed using a biosensor equipped with a glucose oxidase electrode (SBA-40E, Institute of Biology, Shandong Academy of Sciences, China).

### Sample collection and pretreatment

According to their respective growth curves, we separately sampled the logarithmic and stationary phases at 30 h and 60 h (control group) or at 60 h and 130 h (cold induction group), respectively. 10 mL samples were centrifuged at 7,000 × *g* at 4 °C for 5 min, immersed in RNAlock Reagent (Cat: #DP440-01, TIANGEN, China), and stored at −80 °C until use.

### RNA extraction, library preparation and sequencing

Total RNA from *Aurantiochytrium* sp. SD116 was isolated using TRIzol reagent according to the manufacturer’s instructions (Invitrogen, USA). RNA degradation and contamination was monitored on 1% agarose gels. The RNA purity and integrity were checked using a NanoPhotometer spectrophotometer (IMPLEN, CA, USA) and an RNA Nano 6000 assay kit for the Agilent Bioanalyzer 2100 system (Agilent Technologies, CA, USA), respectively. The RNA concentration was measured using a Qubit RNA Assay Kit with a Qubit 2.0 Fluorometer (Life Technologies, CA, USA).

A total amount of 3 μg of RNA per sample was used as the input material for the RNA sample preparations. Sequencing libraries were generated using the NEBNext Ultra^TM^ RNA Library Prep Kit for Illumina (NEB, Ipswich, MA, USA) following the manufacturer’s recommendations. Index codes were added to attribute the sequences to each sample. Briefly, mRNA was purified from total RNA using poly-T oligo-attached magnetic beads. Fragmentation was carried out using divalent cations at an elevated temperature in NEBNext First Strand Synthesis Reaction Buffer (5×). First strand cDNA was synthesized using random hexamer primers and M-MuLV Reverse Transcriptase (RNase H^−^). Second strand cDNA synthesis was subsequently performed using DNA polymerase I and RNase H. The remaining overhangs were converted into blunt ends through exonuclease and polymerase activities. After the 3′ ends of the DNA fragments were adenylated, NEBNext adaptors with a hairpin loop structure were ligated to prepare for hybridization. To preferentially select cDNA fragments of 150–200 bp in length, the library fragments were purified using the AMPure XP system (Beckman Coulter, Beverly, USA). Then, 3 μL of USER enzyme (NEB, USA) was used with size-selected, adaptor-ligated cDNA at 37 °C for 15 min, followed by 5 min at 95 °C before PCR. Then, PCR was performed with Phusion High-Fidelity DNA polymerase, Universal PCR primers, and an index (X) primer. At last, the PCR products were purified (AMPure XP system) and the library quality was assessed using the Agilent Bioanalyzer 2100 system.

The index-coded samples were clustered using a cBot Cluster Generation System and the TruSeq PE Cluster Kit v3-cBot-HS (Illumina) according to the manufacturer’s instructions. After cluster generation, the library preparations were sequenced on an Illumina Hiseq 2000 platform, and paired-end reads were generated.

### RNA-seq data analyses

The raw data (raw reads) in fastaq format were first processed using in-house perl scripts. During this step, the clean data (clean reads) were obtained by removing low-quality reads and reads containing adapters or poly-N sequences from the raw data. At the same time, Q20, Q30, the GC-content, and the sequence duplication levels of the clean data were calculated. All the downstream analyses were based on the clean, high-quality data.

The transcriptome was assembled using Trinity^52^. Min_kner_cov was set to 2 by default and all other parameters were set default. For transcriptome annotation, the final sequences were queried against the NCBI non-redundant protein sequences (Nr) and NCBI nucleotide sequences (Nt) using NCBI blast 2.2.28+ with a cut off E-value ≤ 10^−5^. Sequences with significant matches were annotated using the Blast2GO platform^53^. Additional annotations were obtained from the Kyoto Encyclopedia of Genes and Genomes (KEGG), Protein family (Pfam), Clusters of Orthologous Groups of proteins (KOG/COG) and Swiss-Prot databases.

The gene expression levels were estimated using RSEM^54^ for each sample. Briefly, the clean data were mapped back onto the assembled transcriptome, and then the transcript abundances were calculated as reads per kilobase of exon model per million mapped reads (RPKM). Differential expression analysis (fold changes) and related statistical computations of the two tested conditions were conducted using the DESeq R package (1.10.1). The resulting P-values were adjusted using Benjamini’s and Hochberg’s approach for controlling the false discovery rate. Genes with an adjusted P-value < 0.05 found using DESeq were classified as differentially expressed.

Gene Ontology (GO) enrichment analysis of differentially expressed genes (DEGs) was conducted using the GOseq R package^55^. Next, the KOBAS^56^ software was used to test the statistical enrichment of differentially expressed genes in the KEGG pathways.

### Quantitative real-time PCR (qRT-PCR) validation

Some genes identified in the transcriptome sequencing analysis were further validated and quantified by real-time PCR (qRT-PCR). The primer sequences (Table S2) were designed with Primer Premier 5 according to the transcriptome sequencing data. The total RNA was extracted from the same samples using a Thermo Scientific GeneJET RNA Purification kit (#K0731), as in the Illumina sequencing step. After DNase-mediated DNA degradation, the RNA concentration was determined and 2 μg of total RNA was reverse transcribed in a 20-μl reaction volume using the TIANScript reverse transcription Kit (Tiangen).

qRT-PCR was performed in a LightCycler R480 Real-time Detection System (Roche). The reaction was carried out in a 20-μl reaction volume using the FastStart Universal SYBR Green Master (ROX) according to the manufacturer’s protocol. A melting curve analysis of the amplification products at the end of each reaction was performed to confirm the specificity of amplification. To quantify the transcription of each gene, the copy number was determined by generating a standard curve using a serial 10-fold dilution of the targeted PCR product inserted into the pMD^TM^ 19-T vector (TaKaRa Bio Group). For sample normalization, 18S rRNA was used as an internal standard. All of the reactions were performed in triplicate, and the data were normalized using the average for the internal standard.

### Lipid extraction and fatty acid composition analysis

The total lipid extraction was performed according to our previous method^57^. Briefly, each harvested sample was extracted using chloroform/methanol (2:1, v/v) at room temperature. The lipid extract was dried over anhydrous Na_2_SO_4_, and the solvent was removed by evaporation. The extracted lipid was weighed. Afterward, fatty acid methyl esters (FAMEs) were prepared according to the method described by Song *et al*.^58^.

## Additional Information

**How to cite this article**: Ma, Z. *et al*. Transcriptome and gene expression analysis of DHA producer *Aurantiochytrium* under low temperature conditions. *Sci. Rep*. **5**, 14446; doi: 10.1038/srep14446 (2015).

## Supplementary Material

Supplementary Information

## Figures and Tables

**Table 1:** Fatty Acid Composition of *Aurantiochytrium* sp. SD116 in different samples.

Sample	Lipid content (%)	Fatty acid composition (%)		
		C16:0	DPA(C22:5, n-6)	DHA(C22:6, n-3)
A2530	22.37 ± 1.62	40.532 ± 0.410	8.175 ± 0.316	43.100 ± 1.077
A2560	57.71 ± 3.05	43.523 ± 0.870	8.081 ± 0.204	42.485 ± 0.712
A1560	23.97 ± 2.62	38.666 ± 0.656	8.270 ± 1.082	47.642 ± 0.497
A15130	55.00 ± 1.58	33.38 ± 0.470	9.269 ± 0.729	52.478 ± 1.140

a, the data of every group comes from four replicates; sample name contains temperature and time information (e.g. A2530 means samples cultured for 30 hours at 25 °C).

**Table 2:** Summary of draft reads of samples by Illumina deep sequencing.

Sample name^a^	Raw Reads	Clean Reads	Clean Bases	Error(%)	Q20(%)	Q30(%)	GC Content(%)
A2530	31,374,017	29,587,068	2.96G	0.04	96.68	90.25	52.64
A2560	36,542,482	34,595,077	3.46G	0.04	96.93	90.92	52.71
A1560	33,083,258	26,317,734	2.63G	0.04	96.92	90.57	52.60
A15130	29,402,238	27,737,334	2.78G	0.04	96.74	90.40	52.52

**Table 3:** Distributions of unigenes in different public databases.

Annotated in Databases	Number of Unigenes	Percentage (%)
Annotated in NR	9211	30.92
Annotated in NT	2335	7.84
Annotated in KO	4116	13.81
Annotated in SwissProt	7734	25.96
Annotated in PFAM	11144	37.41
Annotated in GO	11419	38.34
Annotated in KOG	6113	20.52
Annotated in all Databases	980	3.29
Annotated in at least one Database	13245	44.47
Total Unigenes	29783	100

**Table 4:** Functional classification of the up-regulated /down-regulated genes identified in different groups.

Functional category	Number of genes up/down-regulated in different groups			
	A	B	C	D
RNA processing and modification	20/3	1/6	17/15	6/25
Chromatin structure and dynamics	0/0	3/2	6/18	5/16
Energy production and conversion	14/11	22/7	21/57	37/60
Cell cycle control, cell division, chromosome partitioning	15/1	6/2	5/22	5/26
Amino acid transport and metabolism	14/13	13/10	18/56	22/54
Nucleotide transport and metabolism	3/2	3/2	2/17	2/18
Carbohydrate transport and metabolism	5/3	15/4	11/49	13/45
Coenzyme transport and metabolism	2/0	3/0	6/14	6/13
Lipid transport and metabolism	20/17	19/11	42/69	43/67
Translation, ribosomal structure and biogenesis	19/11	2/1	66/10	23/13
Transcription	8/6	5/8	26/17	23/13
Replication, recombination and repair	5/2	4/4	4/41	6/35
Cell wall/membrane/envelope biogenesis	10/3	2/6	10/14	8/19
Cell motility	4/2	0/0	1/5	0/4
Posttranslational modification, protein turnover, chaperones	17/13	18/13	31/70	39/72
Inorganic ion transport and metabolism	21/11	9/12	12/39	19/38
Secondary metabolites biosynthesis, transport and catabolism	9/12	10/8	16/33	18/28
General function prediction only	61/25	34/24	77/125	89/139
Function unknown	20/6	8/9	18/35	8/40
Signal transduction mechanisms	57/13	22/22	62/82	58/108
Intracellular trafficking, secretion, and vesicular transport	6/3	2/4	16/23	12/23
Defense mechanisms	6/0	6/2	5/11	6/10
Extracellular structures	1/1	1/0	1/2	1/1
Unamed protein	0/0	0/0	1/0	1/0
Nuclear structure	1/0	0/0	0/0	0/0
Cytoskeleton	35/8	11/17	17/49	13/52

(A) 15 °C 60 h vs 25 °C 30 h; (B) 15 °C 130 h vs 25 °C 60 h; (Comparisons in different temperatures during lipid accumulation).

(C) 25 °C 60 h vs 25 °C 30 h; (D) 15 °C 130 h vs 15 °C 60 h. (Comparisons in different phases under cold induction).

**Table 5:** Relative mRNA expression of 10 selected genes for comparison between different groups, in respect to RNA-seq and real-time PCR.

Gene ID	Gene Name	15 °C 60 h vs 25 °C 30 h		15 °C 130 h vs 25 °C 60 h		25 °C 60 h vs 25 °C 30 h		15 °C 130 h vs 15 °C 60 h	
		Real-time PCR	RNA-seq	Real-time PCR	RNA-seq	Real-time PCR	RNA-seq	Real-time PCR	RNA-seq
comp24814_c0	type I fatty acid synthase	−1.96 ± 0.02	−1.89	0.77 ± 0.14	−0.35	−4 ± 0.06	−1.75	−1.26 ± 0.2	−0.23
comp22062_c0	PUFA subunit A	−1.31 ± 0.05	−1.12	3.49 ± 0.14	1.68	−11.39 ± 0.04	−10.31	−6.59 ± 0.13	−7.52
comp22942_c2	PUFA subunit B	−1.27 ± 0.35	−1.07	1.18 ± 0.26	1.61	−9.58 ± 0.08	−9.5	−7.35 ± 0.21	−6.83
comp19213_c0	PUFA subunit C	−0.82 ± 0.08	−1.22	2.17 ± 0.27	1.4	−11.94 ± 0.19	−10.29	−8.95 ± 0.12	−7.69
comp24283_c0	acetyl−CoA carboxylase	−1.35 ± 0.16	−1.31	−0.48 ± 0.19	1.05	−5.27 ± 0.02	−4.2	−4.08 ± 0.16	−1.85
comp18299_c0	acyl−CoA:diacylglycerol acyltransferase	0.84 ± 0.04	−1.19	1.75 ± 0.28	−0.73	1.1 ± 0.14	0.6	2.14 ± 0.07	1.04
comp17264_c0	glucose−6−phosphate 1−dehydrogenase	−0.67 ± 0.24	−0.31	1.01 ± 0.12	0.2	−1.52 ± 0.006	−0.66	0.17 ± 0.22	−0.17
comp19802_c0	isocitrate dehydrogenase	−1.32 ± 0.13	−0.01	0.64 ± 0.16	0.72	−2.94 ± 0.06	−1.56	−0.98 ± 0.16	−0.85
comp18937_c0	malic enzyme	−1.6 ± 0.07	−0.98	−0.02 ± 0.06	−0.34	−3.99 ± 0.07	−2.88	−2.42 ± 0.14	−2.27
comp24570_c0	histidine kinase	−1.41 ± 0.12	−0.62	5.25 ± 0.19	2.18	−4.4 ± 0.02	−1.62	2.27 ± 0.22	1.15
