# Supplementary material for: Transcriptome and gene expression analysis of DHA producer Aurantiochytrium under low temperature conditions
Source: Sci Rep. 2015 Sep 25;5:14446. doi: 10.1038/srep14446 (PMC4585886; doi:10.1038/srep14446)
Supplement: Supplementary Information [file srep14446-s1.pdf]

Transcriptome and gene expression analysis of DHA producer *Aurantiochytrium* under low temperature conditions

Zengxin Ma, Yanzhen Tan, Guzhen Cui, Yingang Feng, Qiu Cui*, Xiaojin Song*

Supplementary Tables

Table S1 The gene list mentioned in this paper

Gene ID	Gene Name	A (log2)	B (log2)	C (log2)	D (log2)
comp8488_c0	long-chain acyl-CoA synthetase	4.5272	-0.26602	-1.518	-6.3322
comp9858_c0	long-chain acyl-CoA synthetase	3.3229	-1.426	-1.6247	-6.3931
comp23582_c0	long-chain acyl-CoA synthetase	1.3361	0.81704	0.99928	0.46051
comp21778_c0	carnitine o-acyltransferase CPT1	-0.5964	-0.27396	-0.20951	-0.17541
comp13568_c0	carnitine o-acyltransferase CPT2	-1.7940	0.20756	-4.9531	-2.9725
comp20707_c0	lysophosphatidic acid acyltransferase	2.0777	-1.3369	0.18576	-3.2473
comp15661_c0	phosphatidylinositol transfer protein	3.6181	-2.8861	3.1522	-3.3728
comp17284_c0	phosphatidylinositol transfer protein	1.9338	0.96591	-0.61054	-1.5985
comp22739_c0	phosphatidylinositol transfer protein	1.6411	-0.5737	-2.2296	-4.4649
comp20026_c0	phospholipase	-2.9594	0.01428	-3.1513	-0.1977
comp19985_c0	phospholipase	-0.8015	-0.80679	-0.21164	-0.23672
comp8655_c0	sterol 24-C-methyltransferase	0.15266	2.4544	-6.9223	-4.6412
comp20352_c0	cycloartenol synthase	-0.4370	2.3954	-7.549	-4.7362
comp22215_c1	cholesterol transport protein	-0.7083	1.4086	-1.2684	0.82767
comp15636_c0	squalene synthetase	-0.9352	0.9374	-4.1445	-2.2919
comp24570_c0	histidine kinase	-0.6164	2.1805	-1.6246	1.1526
comp22584_c0	histidine kinase	-0.11989	0.9495	1.2044	2.2539
comp23786_c0	tyrosine kinase	3.4722	1.6506	-3.5923	-5.4329
comp22047_c0	tyrosine kinase	3.1486		-4.7277	
comp22542_c0	tyrosine kinase	2.4356	-0.40762	-0.61852	-3.482
comp19709_c0	tyrosine kinase	2.3202	-0.0028695	-0.97792	-3.321
comp18655_c0	cAMP-dependent protein	3.0604	-1.5411	-1.4688	-6.09

	kinase				
comp20704_c0	cAMP-dependent protein kinase	2.2289	-2.5857	0.80292	-4.032
comp20704_c2	cAMP-dependent protein kinase	2.4063	-1.7943	0.7824	-3.4387
comp20035_c0	G protein signaling regulators	4.0181	-0.96178	-3.2746	-8.2765
comp248022_c0	G protein signaling regulators	3.2354	1.2167	-0.073159	-2.1125
comp15887_c0	G protein signaling regulators	1.7594	1.1715	-0.72313	-1.3316
comp21331_c0	Ca^{2+} sensor	4.4460	-0.81405	-3.4539	-8.7312
comp34371_c0	Ca2+/calmodulin-dependent protein kinase	3.1311	0.93995	2.0831	-0.13094
comp23037_c0	Ca3+/calmodulin-dependent protein kinase	3.1290	-1.6792	-0.26391	-5.0904
comp21567_c0	Ca4+/calmodulin-dependent protein kinase	2.0532	2.0484	-0.57406	-0.59925
comp10178_c0	phosphoinsositide 3-kinase	3.0172	0.72196	0.84373	-1.4734
comp20282_c0	phosphoinsositide 3-kinase	2.8901	0.499	1.1019	-1.3085
comp21271_c0	diacylglycerol kinase	3.0954	1.7795	-3.2385	-4.5717
comp21402_c0	hexokinase	-0.7817	-0.2788	-2.982	-2.4998
comp24336_c2	phosphofructokinase	-0.5848	-0.082597	-1.4833	-1.0009
comp22393_c3	Fructose-1,6-bisphosphatase	0.3625	0.012547	0.13561	-0.23445
comp20168_c0	glyceraldehyde 3-phosphate dehydrogenase	1.3883	0.8500	-0.60549	-1.1639
comp15593_c0	6-phosphogluconate dehydrogenase	-0.17146	0.3716	-1.8025	-1.2798
comp17839_c0	ribokinase	0.25684	0.5159	-1.2834	-1.0445
comp12193_c0	ribose 5-phosphate isomerase	0.5563	0.3047	-0.67777	-0.94995
comp25182_c0	succinyl-CoA synthetase	-0.6865	0.2254	-2.0235	-1.1319
comp18922_c0	fumarate hydratase	-0.5120	-0.9384	-0.16484	-0.61194
comp15787_c0	fumarate hydratase	-0.8247	-0.7117	-2.2377	-1.6634
comp17343_c0	citrate synthase	-0.097098	0.5349	-0.73812	-0.12664

(A). 15 ℃ 60h vs 25 ℃ 30h; (B). 15 ℃ 130h vs 25 ℃ 60h; (Comparisons in different temperatures during lipid accumulation)

(C). 25 ℃ 60h vs 25 ℃ 30h; (D). 15 ℃ 130h vs 15 ℃ 60h. (Comparisons in different phases under cold induction)

Table S2 Primers for genes validated by Quantitative real-time PCR (qRT-PCR)

Genes	Sequences
18S_F	TGCCGACTTGCGATTGTTG
18S_R	TTCAGCCTTGCGACCATACT
Fas_F	GCATCTACCACCGTCTTGTTG
Fas_R	GGAGCAGAACCAGTCACCTT
Pfaa_F	AGCCTCCTTGATAGCCTTCTC
Pfaa_R	TCTGGTGCGTGTTCTTGGT
Pfab_F	GTCGGAGTAGGTGGCTTGT
Pfab_R	GCCTTCATCGTCACTGGTAC
Pfac_F	CTGGTGGTGGTGTTGGATG
Pfac_R	GCTGCTTGCGGACATTGT
Acc_F	GAGACCACTTACCGCCTGTT
Acc_R	CGCCAATGAGCACAAGGAAG
Dgat_F	GCGTCAGTTGGTGTAGAGTTG
Dgat_R	TGTCGGTCGGTGGAACTTG
g6pd_F	CCAGAGACTCCTTCGGCATAT
g6pd_R	CCTCCAACACCTCGTCAAGA
Icd_F	GGTTGGTGGCATTGGCATT
Icd_R	CGGAGAGGAGAACACTGGAG
Me_F	AAGCCTGCCAAGAGTTCCA
Me_R	GCCAGTTGTTCACGAGAGTC
Hsk_F	CCGCCACTGATATTCCAATCC
Hsk_R	CCGCACTGACGAAGATGAC

Abbreviations for genes: acc, acetyl-CoA carboxylases; dgat, diacylgycerol acyltransferase; fas, fatty acid synthase; g6pd, gycose-6-phosphate dehydrogenase; hsk, histidine kinase; icd, isocitrate dehydrogenase; me, malic enzyme; pfa A, polyunsaturated fatty acid synthase subunit A; pfa B, polyunsaturated fatty acid synthase subunit B; pfa C, polyunsaturated fatty acid synthase subunit C.

Supplementary Figure

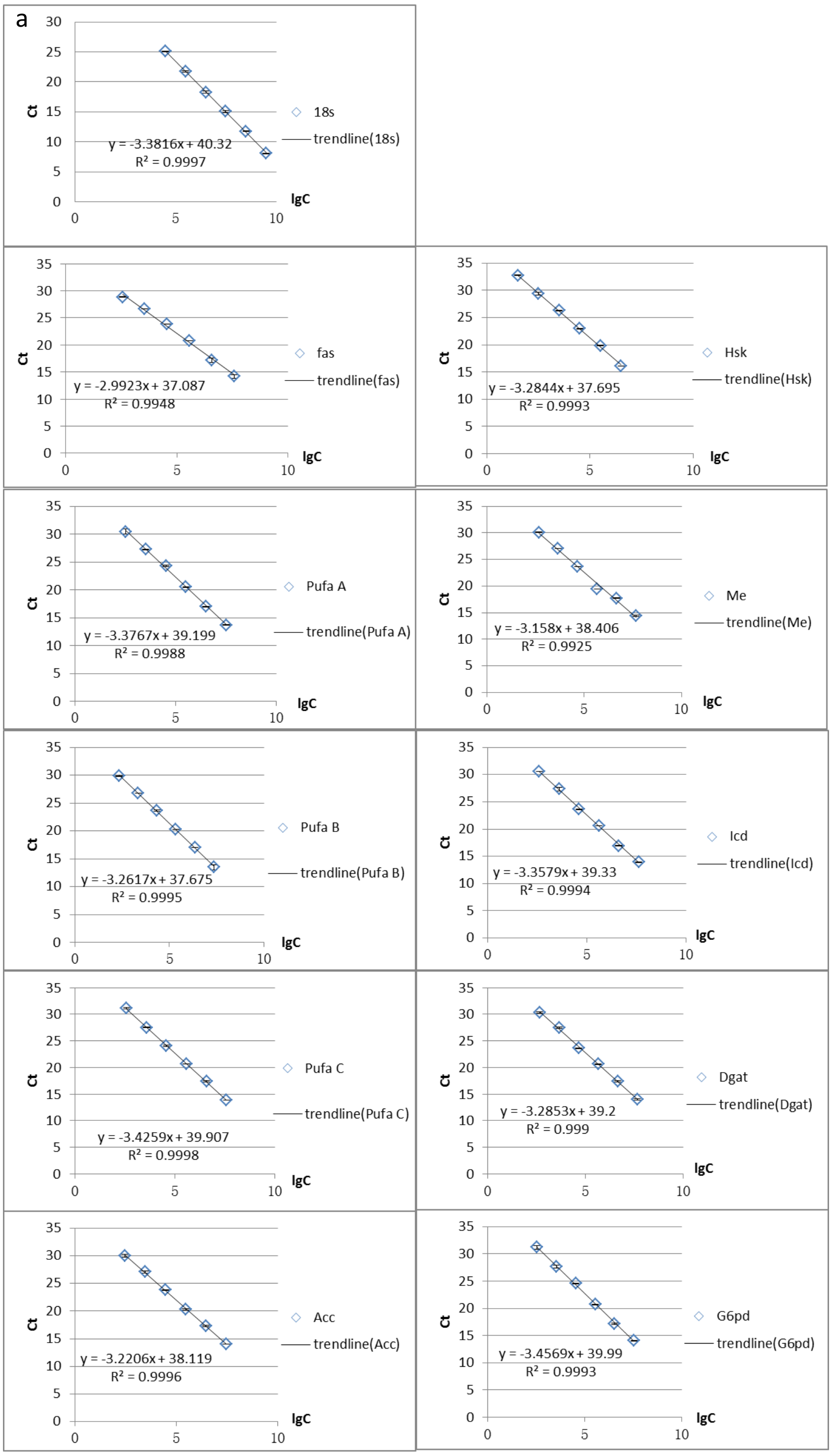

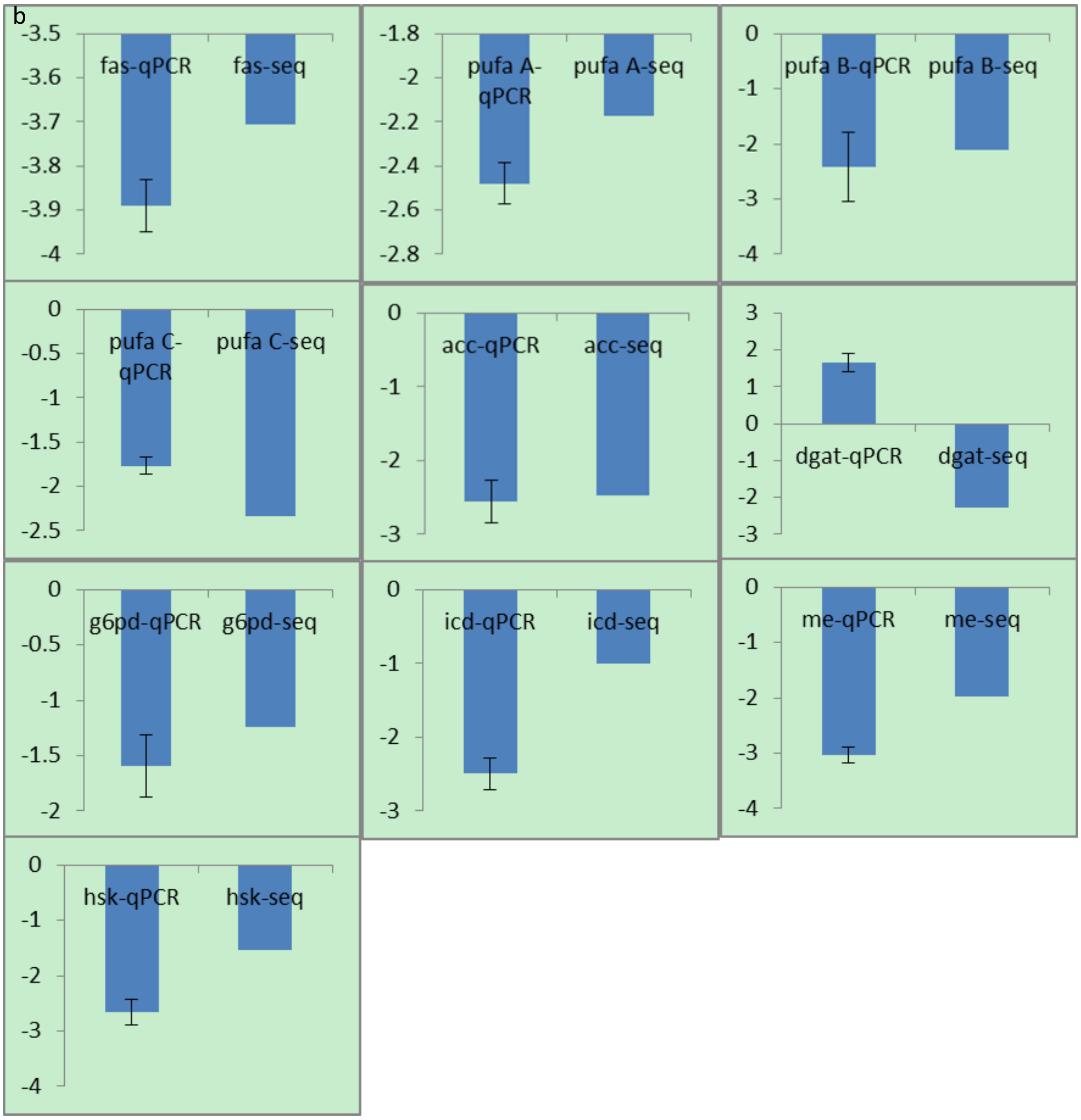

b
fas-qPCR
fas-seq
pufa A-qPCR
pufa A-seq
pufa B-qPCR
pufa B-seq
pufa C-qPCR
pufa C-seq
acc-qPCR
acc-seq
dgat-qPCR
dgat-seq
g6pd-qPCR
g6pd-seq
icd-qPCR
icd-seq
me-qPCR
me-seq
hsk-qPCR
hsk-seq

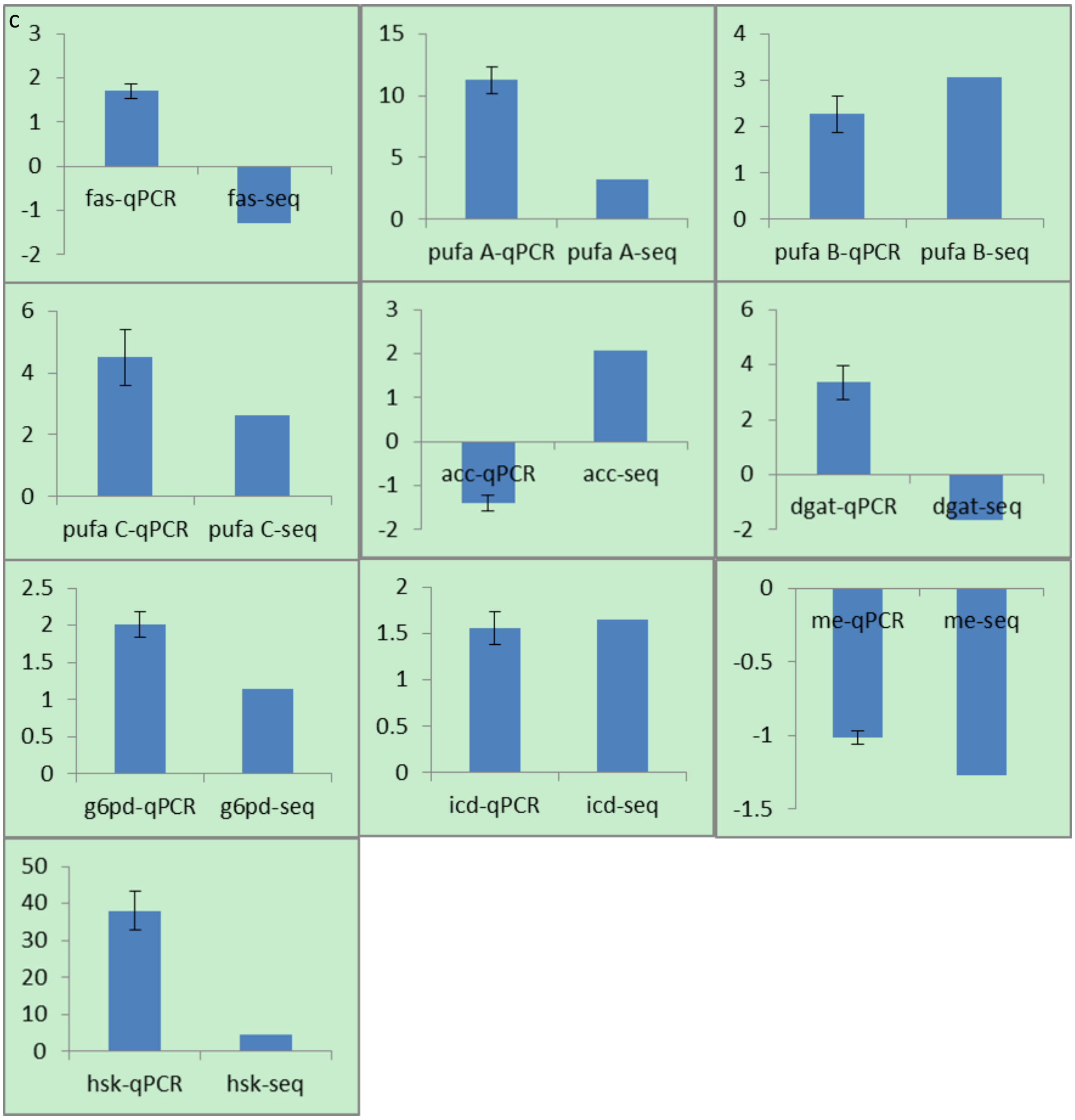
c
fas-qPCR
fas-seq
pufa A-qPCR
pufa A-seq
pufa B-qPCR
pufa B-seq
pufa C-qPCR
pufa C-seq
acc-qPCR
acc-seq
dgat-qPCR
dgat-seq
g6pd-qPCR
g6pd-seq
icd-qPCR
icd-seq
me-qPCR
me-seq
hsk-qPCR
hsk-seq

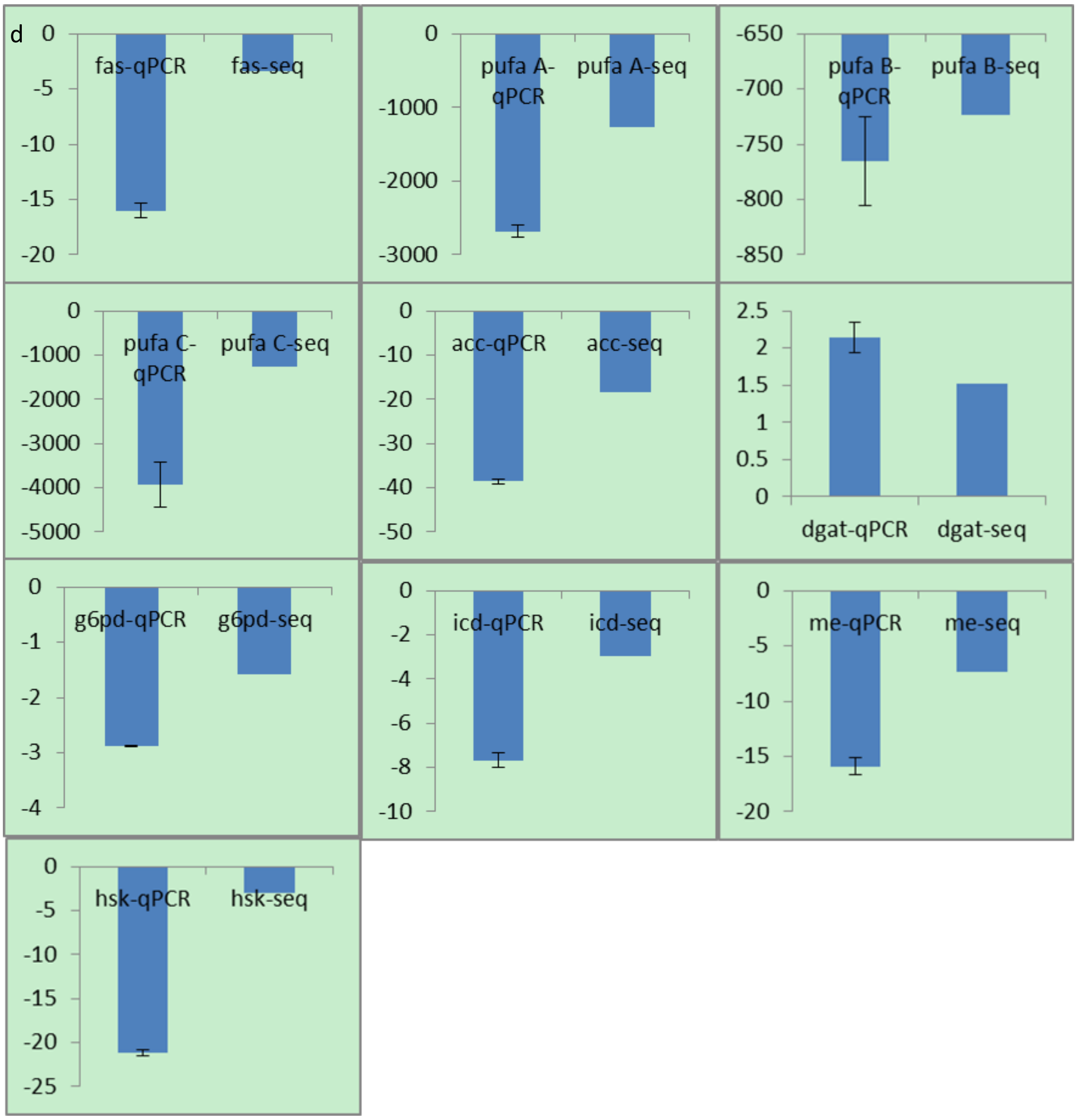
d
fas-qPCR
fas-seq
pufa A-qPCR
pufa A-seq
pufa B-qPCR
pufa B-seq
pufa C-qPCR
pufa C-seq
acc-qPCR
acc-seq
dgat-qPCR
dgat-seq
g6pd-qPCR
g6pd-seq
icd-qPCR
icd-seq
me-qPCR
me-seq
hsk-qPCR
hsk-seq

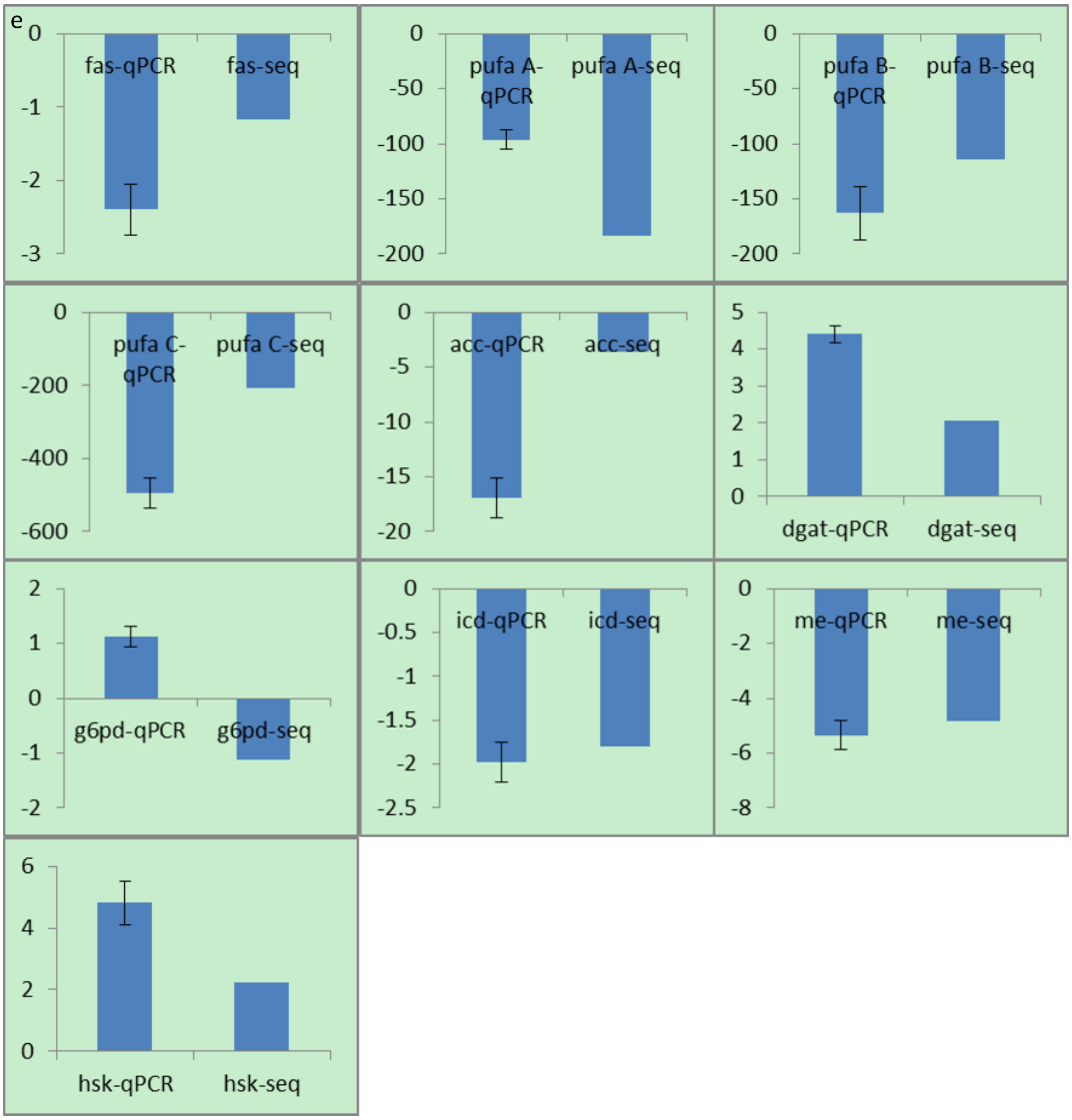

Supplementary Figure S1 Changes in gene expression levels confirmed by qRT-PCR and the standard curve. a) standard curve of each gene for qRT-PCR test; b) 15 °C 60h vs 25 °C 30h; c) 15 °C 130h vs 25 °C 60h; d) 25 °C 60h vs 25 °C 30h; e) 15 °C 130h vs 15 °C 60h.

Abbreviations for genes: acc, acetyl-CoA carboxylases; dgat, diacylgycerol acyltransferase; fas, fatty acid synthase; g6pd, gycose-6-phosphate dehydrogenase; hsk, histidine kinase; icd, isocitrate dehydrogenase; me, malic enzyme; pufa A, polyunsaturated fatty acid synthase subunit A; pufa B, polyunsaturated fatty acid synthase subunit B; pufa C, polyunsaturated fatty acid synthase subunit C.
